# Dance Interventions Improve Quality of Life and Related Psychological Factors Among Breast Cancer Survivors: A Systematic Review and Meta‐Analysis

**DOI:** 10.1002/pchj.70091

**Published:** 2026-03-31

**Authors:** Tingting Chen, Lei Jiang, Xiaohong Xu, Changhao Jiang

**Affiliations:** ^1^ Faculty of Dance Education Beijing Dance Academy Beijing China; ^2^ Institute of Physical Education and Training Capital University of Physical Education and Sports Beijing China; ^3^ School of Kinesiology and Health Capital University of Physical Education and Sports Beijing China; ^4^ Beijing Key Lab of Physical Fitness Evaluation and Tech Analysis Capital University of Physical Education and Sports Beijing China

**Keywords:** breast cancer, dance interventions, meta‐analysis, psychological outcome, quality of life

## Abstract

This systematic review and meta‐analysis examined evidence from randomized controlled trials (RCTs) on the effects of dance interventions on quality of life (QoL) in women with breast cancer. Twelve RCTs were included, and pooled effect sizes were calculated as standardized mean differences (*SMD*s) with 95% confidence intervals (CIs). Compared with controls, dance interventions significantly improved QoL (*SMD* [95% CI] = 0.62 [0.20, 1.05], *p* = 0.004) and reduced depressive symptoms (*SMD* [95% CI] = −0.81 [−1.50, −0.12], *p* = 0.02). Subgroup analyses indicated that these effects were primarily driven by dance‐based physical activity (DPA), particularly in interventions lasting ≥ 12 weeks. No overall effects were found for fatigue, pain, or body image, although modest fatigue reductions were observed in DPA programs ≥ 9 weeks. Dance interventions integrating physical activity, emotional expression, and social interaction may enhance QoL and psychological well‐being in women with breast cancer, although evidence for improvements in physical symptoms remains limited. Further research should standardize intervention protocols and assess long‐term outcomes.

## 1 Introduction

Breast cancer is the most commonly diagnosed malignancy among women, accounting for approximately 26% of all female cancer cases worldwide (Sung et al. 2021). It remains the leading cause of cancer‐related mortality among women aged 35 to 54 years (Barzaman et al. 2020; Sung et al. 2021). According to GLOBOCAN data from 2020, breast cancer surpassed lung cancer as the most prevalent cancer in women, with around 2.3 million new cases and 685,000 deaths reported globally (Sung et al. 2021). Standard treatments such as chemotherapy, radiotherapy, surgery, immunotherapy, and hormonotherapy have significantly improved survival rates (Waks and Winer 2019). However, these therapies often contribute to a substantial decline in health‐related quality of life (HRQoL) among survivors, necessitating interventions to address this issue.

Health‐related quality of life extends beyond the absence of disease, encompassing a holistic state of physical, mental, and social well‐being (Howard‐Anderson et al. 2012). An optimal QoL is characterized by robust physical functioning, emotional resilience, and fulfilling social connections. However, a breast cancer diagnosis, compounded by the challenges of demanding treatment regimens, profoundly disrupts these aspects of well‐being. Survivors often contend with persistent depression and anxiety, driven by fears of disease recurrence, diminished physical abilities, and an uncertain future (Howard‐Anderson et al. 2012; Carreira et al. 2018). These psychological and emotional stressors, whether acting independently or synergistically, can lead to marked declines in QoL (Fortin et al. 2021). Consequently, contemporary cancer care has increasingly prioritized non‐pharmacological interventions to help survivors more effectively manage these challenges and enhance their overall HRQoL.

Physical activity has emerged as a promising strategy for enhancing QoL among breast cancer survivors. Research consistently demonstrates that regular physical activity benefits survivors irrespective of their clinical stage or treatment history (Bedillion et al. 2019). Among these interventions, dance stands out as a multifaceted approach that integrates physical movement with mental and social engagement, uniquely addressing the complex needs of this population (Chan et al. 2020). Unlike traditional forms of exercise, dance enables participants to release physical tension and express emotions through spontaneous, rhythmic movement. For example, a 12‐month Hula dance program significantly enhanced vigor, as assessed by the Profile of Mood States (POMS) (Loo et al. 2019). Similarly, a 24‐week Greek traditional dance led to a 19.9% improvement in physical function, a 36.3% increase in life satisfaction, and a 35% reduction in depressive symptoms among breast cancer survivors (Kaltsatou et al. 2011). Furthermore, a 13‐week dance/movement therapy (DMT) program produced significant improvements in breast cancer‐specific HRQoL, as measured by the Functional Assessment of Cancer Therapy‐Breast (FACT‐B), with sustained effects observed at 26 weeks (Sandel et al. 2005). Collectively, these findings highlight the potential of dance to deliver diverse psychological and physical benefits, helping survivors adopt a more adaptive and resilient perspective on cancer treatment as a life challenge (Teixeira‐Machado et al. 2019).

Dance interventions for breast cancer survivors can be classified into two conceptually distinct categories based on therapeutic orientation. The first is DMT, defined by the American Dance Therapy Association as “the psychotherapeutic use of movement to promote emotional, social, cognitive, and physical integration of the individual for the purpose of improving health and well‐being” (Chen et al. 2022). DMT is delivered by a trained therapist and centers on the therapeutic relationship, using guided movement exploration, interpersonal attunement, and reflective discussion to facilitate emotional expression and psychological processing. Typically conducted in group settings, DMT aims to promote psychological resilience through embodied emotional experience and relational interaction (Ho et al. 2020; Millman et al. 2021). The second category comprises dance‐based physical activity (DPA), including forms such as belly dance, square dance, Greek traditional dance, and ballroom dance (Douka et al. 2019). These programs are instructor‐led and primarily emphasize structured movement practice, rhythmic coordination, and group participation within a standardized session format (e.g., warm‐up, main activity, cool‐down). Unlike DMT, DPA does not involve therapist‐guided emotional processing; its benefits are presumed to arise from physical exercise, rhythmic entrainment, and social engagement. This classification reflects a therapeutic‐orientation framework commonly used in rehabilitation research, distinguishing interventions by mechanism rather than dance genre.

Although both DMT and DPA have shown psychological benefits in breast cancer populations, prior reviews have relied on narrative synthesis and rarely examined whether therapeutic orientation moderates outcomes (Bradt et al. 2015). The present meta‐analysis therefore evaluates dance interventions at two levels: estimating overall efficacy and testing therapeutic orientation as a potential moderator. Because both forms share core embodied features—movement, rhythm, and interpersonal coordination (Sevdalis and Keller 2009)—pooled analysis was conducted to assess general effects, while subgroup analyses by intervention type and duration addressed potential heterogeneity. The pooled analysis estimates higher‐order effects of dance‐based behavioral interventions, and subgroup comparisons examine whether mechanism moderates efficacy. Subgroup analysis by specific dance genres was not performed, as individual genres were represented by too few trials and did not consistently map onto distinct mechanisms, which could introduce spurious heterogeneity. By synthesizing RCTs comparing dance interventions with active or inactive controls, this study aims to quantify pooled effects on health‐related quality of life and psychological outcomes, determine whether DMT and DPA differ in efficacy, and inform intervention selection and future research.

## 2 Materials and Methods

### 2.1 Search Strategy

Five electronic databases (PubMed, EBSCO, MEDLINE, Cochrane, and PsycINFO) were searched from inception through November 2024. The search terms included (“breast cancer” or “breast neoplasms”) and (“dance” or “dancing” or “creative movement”). References in the identified studies were also reviewed to identify additional studies that might have been missed. Two independent investigators screened the studies and cross‐checked for consistency. The results were exported to EndNote X9, organized by database, and then to Excel for further screening.

### 2.2 Inclusion/Exclusion Criteria

Studies were eligible if they involved women diagnosed with breast cancer at any stage of treatment or survivorship, without restrictions on demographic or socioeconomic characteristics. Interventions had to involve DMT or DPA and report at least one validated physical or psychological outcome. Eligible studies were randomized controlled trials (RCTs), peer‐reviewed, and published in English. Studies were excluded if they lacked a control or comparison group, were qualitative in design, or were reviews, commentaries, book chapters, or dissertations. When multiple publications reported data from the same cohort, the report with the most complete dataset was retained to avoid duplication.

### 2.3 Study Screening and Data Extraction

Two researchers independently and blindly screened studies using a two‐step process. First, they reviewed titles and abstracts, categorizing them as relevant, possibly relevant, or irrelevant. Next, they thoroughly reviewed articles classified as relevant or possibly relevant to determine their eligibility. Disagreements were resolved through consensus. From each included study, the following information was extracted: authors, publication year, study design, participant characteristics (including clinical stage, mastectomy status, and adjuvant treatment), intervention protocol, outcome measures, and reported results.

### 2.4 Quality Assessment

The methodological quality of the included studies was evaluated using the Physiotherapy Evidence Database (PEDro) scale (Cashin and McAuley 2020). The PEDro scale comprises 11 items, with the total score calculated by summing the ratings of items 2–11, resulting in a range from 0 to 10. Higher scores indicate superior methodological quality. Study quality was categorized as excellent (9–10 points), good (6–8 points), fair (4–5 points), or poor (less than 4 points).

### 2.5 Data Analysis

A meta‐analysis of the included RCTs was conducted using Review Manager 5.3 software. Data extracted from the included studies consisted of mean (*M*), sample size (*N*), and standard deviation (SD), which served as the primary inputs for ES calculations (Healy et al. 2018). In cases where these data were unavailable, *F*‐values, *t*‐values, and/or *p*‐values were extracted and utilized for ES estimation (Gurevitch et al. 2018). For studies reporting multiple intervention groups, only the group corresponding to the dance intervention was extracted, and other groups were excluded. The standardized mean difference (SMD) and corresponding 95% confidence intervals (CIs) were calculated for each study. The SMD was defined as the difference in pre‐posttest mean changes between the dance group and the control group, divided by the pooled standard deviation of the measurements, following the methods described by Sonuga‐Barke et al. (2013). A random‐effects model was applied to estimate pooled ES, as it provides a more conservative and generalizable estimate of ES across studies (Gurevitch et al. 2018; Sonuga‐Barke et al. 2013). Forest plots were generated to display the SMD and corresponding CIs for individual studies, along with the overall pooled estimate. Heterogeneity among the studies was assessed using the *I* ^*2*^ statistic, as recommended by Mattle et al. (2020), with thresholds of 25%, 50%, and 75% used to categorize low, moderate, and high heterogeneity, respectively.

## 3 Results

### 3.1 Search Outcomes and Study Characteristics

**FIGURE 1:**
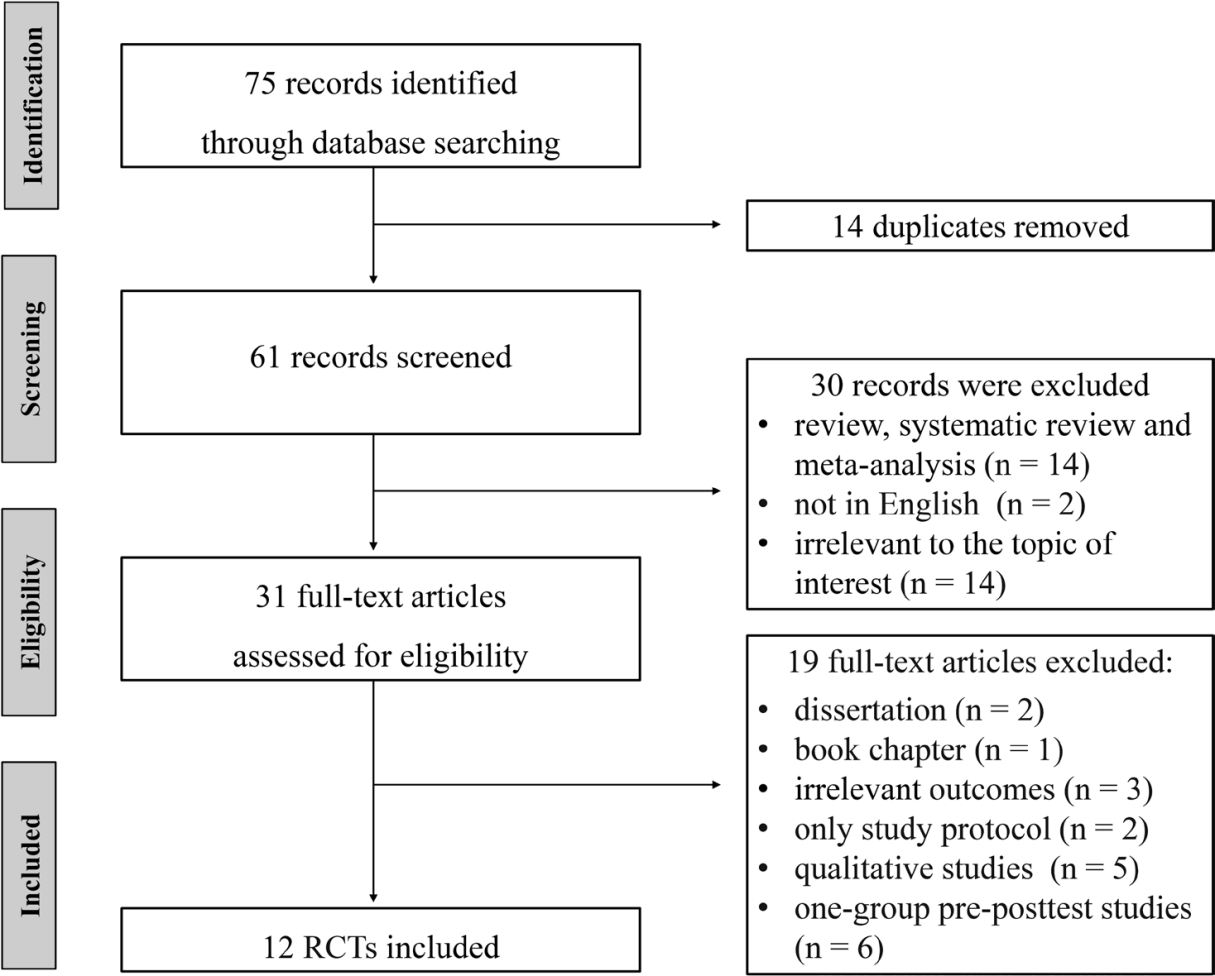
PRISMA 2009 flow diagram.

Seventy‐five records were initially identified, of which 14 duplicates were removed. After title and abstract screening, 30 articles were excluded for not meeting the selection criteria. The remaining 31 articles underwent full‐text assessment (Figure 1). Ultimately, 12 RCTs published between 2000 and 2023 met the predefined criteria and were included in the analysis (Table 1).

**TABLE 1:** Characteristics of 12 RCTs.

Study	Country	Participants	Clinical stage	Mastectomy	Adjuvant treatment	Intervention	Period and frequency	Outcomes of interest	Measures	Pretest vs. posttest (M ± SD)
Boing et al. (2018)	Brazil	EG: *n* = 8, age = 54.1 ± 7.6	Stage I–III	EG: *n* = 7	53.3% Hormonal therapy	Belly dance vs. No intervention	60 min per session	HRQoL	EORTC QLQ‐BR23	EG: 44.0 ± 26.0 vs. 65.0 ± 21.0
		CG: *n* = 11, age = 54.1 ± 7.6		CG: *n* = 7	26.7% Chemotherapy		2 sessions per week	Depression	BDI	CG: 65.0 ± 24.0 vs. 68.0 ± 17.0
										EG: 19.0 ± 10.0 vs. 11.0 ± 10.0
										CG: 8.0 ± 7.0 vs. 11.0 ± 7.0
					20.0% Radiotherapy		12 weeks	Fatigue	PFS	EG: 5.0 ± 3.0 vs. 4.0 ± 2.0
										CG: 4.0 ± 2.0 vs. 5.0 ± 2.0
Boing et al. (2023)	Brazil	EG: *n* = 25, age = 55.0 ± 9.9	Stage I–III	EG: *n* = 8	100% Hormonal therapy	Belly dance vs. Mat Pilates VS. Educational activities	60 min per session	Depression	BDI	EG: 12.3 ± 1.7 vs. 9.9 ± 1.5
		CG1: *n* = 25, age = 54.3 ± 10.4		CG1: *n* = 13			3 sessions per week	Fatigue	FACT‐F	
										CG: 11.4 ± 2.0 vs. 10.1 ± 2.4
										EG: 38.7 ± 2.6 vs. 43.3 ± 1.3
		CG2: *n* = 24, age = 56.8 ± 11.2		CG2: *n* = 7			16 weeks			CG: 40.5 ± 1.8 vs. 43.7 ± 1.6
Carminatti et al. (2019)	Brazil	EG: *n* = 8, age = 54.6 ± 8.3	Stage I–III	EG: *n* = 7	53.3% Hormone therapy	Belly dance vs. No intervention	60 min per session	Body Image	BIABCQ	EG: 38.0 ± 3.0 vs. 31.0 ± 2.0
		CG: *n* = 11, age = 54.6 ± 8.3		CG: *n* = 7	26.7% Chemotherapy		2 session per week	Self‐esteem	RSES	CG: 27.0 ± 3.0 vs. 27.0 ± 2.0
										EG: 29.0 ± 1.0 vs. 32.0 ± 2.0
					20.0% Radiotherapy		12 weeks			CG: 32.0 ± 1.0 vs. 32.0 ± 1.0
Dibbell‐Hope (2000)	USA	EG: *n* = 14, age = 35–80	Stage I–II	81% Surgery	21% Chemotherapy	DMT vs. Wait list control	180 min per session	Body Image	BWB	EG: NR vs. 4.0 ± 1.0
										CG: NR vs. 4.0 ± 1.3
		CG: *n* = 15, age = 35–80			19% Radiotherapy		1 session per week		SCL‐90	EG: NR vs. 0.7 ± 0.8
								Depression	POMS	CG: NR vs. 0.6 ± 0.5
								Fatigue	BWB	EG: NR vs. 6.4 ± 4.6
							6 weeks	Self‐esteem		CG: NR vs. 9.9 ± 5.7
										EG: NR vs. 3.3 ± 0.8
										CG: NR vs. 3.6 ± 0.6
He et al. (2022)	China	EG: *n* = 88, age = 48.0 ± 8.6	Stage I–III	EG: *n* = 77	51% Chemotherapy	Square dance vs. Health consultation	30 min per session	HRQoL	FACT‐B	EG: 97.7 ± 11.9 vs. 102.0 ± 15.1
		CG: *n* = 88, age = 48.3 ± 10.0		CG: *n* = 78	78% Hormone therapy		5 sessions per week			CG: 96.6 ± 13.4 vs. 92.4 ± 15.5
							16 weeks			
Hiansdt et al. (2021)	Brazil	EG: *n* = 11, age = 55.7 ± 7.3	Stage I–III	EG: *n* = 6	66.7% Hormone therapy	DMT vs. No intervention	60 min per session	Sleep	PSQI	EG: 5.0 ± 1.7 vs. 6.4 ± 2.8
		CG: *n* = 10, age = 54.8 ± 9.6		CG: *n* = 5	9.5% Chemotherapy		2 session per week	Pain	VAS	CG: 5.3 ± 2.9 vs. 6.7 ± 3.1
					4.8% Radiotherapy		12 weeks			EG: 3.8 ± 3.5 vs. 4.6 ± 3.2
										CG: 3.2 ± 3.4 vs. 2.9 ± 3.7
Ho et al. (2016)	Hong Kong, China	EG: *n* = 69, age = 48.6 ± 7.7	Stage 0–III	EG: *n* = 27	79% Chemotherapy	DMT vs. Wait list control	90 min per session	HRQoL	FACT‐B	EG: 97.1 ± 18.8 vs. 98.9 ± 20.0
										CG: 97.9 ± 18.1 vs. 97.8 ± 18.4
		CG: *n* = 70, age = 49.1 ± 8.7		CG: *n* = 30	100% Radiotherapy		2 sessions per week	Depression	HADS	EG: 5.5 ± 3.4 vs. 5.5 ± 3.7
								Fatigue	BFI	CG: 5.8 ± 4.0 vs. 5.5 ± 3.4
								Pain	BPI	EG: 4.6 ± 2.3 vs. 4.2 ± 2.2
							3 weeks	Sleep	PSQI	CG: 4.4 ± 2.3 vs. 4.0 ± 2.4
										EG: 2.9 ± 2.2 vs. 2.8 ± 2.1
										CG: 2.3 ± 2.2 vs. 2.9 ± 2.3
										EG: 7.5 ± 3.9 vs. 7.1 ± 3.9
										CG: 7.3 ± 4.2 vs. 7.5 ± 4.2
Kaltsatou et al. (2011)	Greece	EG: *n* = 14, age = 56.6 ± 4.2	Post‐treatment survivorship (> 3 months)	NR	Received chemotherapy and radiotherapy (NR for percentage)	Greek traditional dance vs. Wait list control	60 min per session	HRQoL	LSI	EG: 33.3 ± 4.9 vs. 45.4 ± 4.9
		CG: *n* = 13, age = 57.1 ± 4.1					3 sessions per week	Depression	BDI	CG: 33.3 ± 4.0 vs. 33.3 ± 4.0
										EG: 25.5 ± 1.7 vs. 16.5 ± 1.7
							24 weeks			CG: 22.3 ± 7.7 vs. 22.3 ± 7.7
Leite et al. (2021)	Brazil	EG: *n* = 18, age = 55.9 ± 10.9	Stage 0–III	EG: *n* = 5	100% Hormone therapy	Belly dance vs. Mat pilates vs. Educational activities	60 min per session	Depression	BDI	EG: 12.1 ± 2.0 vs. 9.7 ± 1.9
		CG1: *n* = 18, age = 55.9 ± 10.9		CG1: *n* = 10			3 sessions per week	Self‐esteem	EAR	CG: 10.8 ± 2.1 vs. 10.8 ± 2.0
										EG: 32.1 ± 1.3 vs. 33.3 ± 1.0
		CG2: *n* = 16, age = 55.9 ± 10.9		CG2: *n* = 4			16 weeks			CG: 31.9 ± 1.4 vs. 32.2 ± 1.1
Pisu et al. (2017)	USA	EG: *n* = 15, age = 57.9 ± 9.3	Post‐treatment survivorship (> 3 months)	EG: *n* = 6	Received chemotherapy and radiotherapy (NR for percentage)	Ballroom dance vs. Wait list control	45 min per session	HRQoL	SF‐36	EG: 60.2 ± 17.9 vs. 65.1 ± 17.7
		CG: *n* = 16, age = 57.9 ± 9.3		CG: *n* = 7			10 session per week	Pain		CG: 61.7 ± 19.4 vs. 59.9 ± 17.0
										EG: 79.2 ± 14.0 vs. 73.7 ± 13.0
							12 weeks			CG: 82.2 ± 15.6 vs. 78.9 ± 18.5
Rubio et al. (2022)	Colombia	EG: *n* = 31, age = 57.0 ± 8.7	Post‐treatment survivorship (> 6 months)	NR	Completed treatment (type NR)	DMT vs. Wait list control	60 min per session	HRQoL	EORTC QLQ‐C30	EG: 86.1 ± 20.9 vs. 86.4 ± 20.0
										CG: 90.7 ± 13.4 vs. 89.9 ± 13.8
		CG: *n* = 33, age = 55.8 ± 10.3					3 session per week	Fatigue		EG: 21.8 ± 16.2 vs. 18.7 ± 17.5
										CG: 13.0 ± 16.6 vs. 15.3 ± 15.0
										EG: 22.7 ± 19.8 vs. 22.7 ± 19.8
							8 weeks	Pain		CG: 12.6 ± 23.4 vs. 12.6 ± 23.4
Sandel et al. (2005)	USA	EG: *n* = 19, age = 59.7 ± 9.8	Post‐treatment survivorship (> 6 months)	EG: *n* = 10	14% Chemotherapy	DMT vs. Wait list control	40–60 min per session	HRQoL	FACT‐B	EG: 102.0 ± 15.8 vs. 116.7 ± 16.9
		CG: *n* = 16, age = 59.5 ± 13.3		CG: *n* = 6	8% Radiotherapy		2 sessions per week	Self‐esteem	SF‐36	CG: 108.1 ± 17.3 vs. 106.1 ± 22.3
										EG: 19.4 ± 6.7 vs. 15.2 ± 6.1
							12 weeks			CG: 19.7 ± 7.1 vs. 16.9 ± 5.2

Abbreviations: BDI = Beck Depression Inventory; BFI = Brief Fatigue Inventory; BIABCQ = Body Image After Breast Cancer Questionnaire; BPI = Brief Pain Inventory; BWB = Borscheid‐Walster‐Bohrnstedt Body‐Image Scale; CG = control group; EG = experimental group; EORTC‐FA12 = European Organization for Research and Treatment of Cancer Quality‐of‐Life Fatigue Module; EORTC QLQ‐C30 = European Organization for Research and Treatment of Cancer Quality‐of‐Life Core Questionnaire; FACT‐F = Functional Assessment of Cancer Therapy‐Fatigue instrument; HADS = Hospital Anxiety and Depression Scale; LSI = Life Satisfaction Inventory; NR = not reported in the original study; PFS = Piper Fatigue Scale; POMS = Profile of Mood States; PSQI = Pittsburgh Sleep Quality Index; RSES = Rosenberg Self‐Esteem Scale; SCL‐90 = Symptom Checklist 90; SF‐36 = Short Form health survey‐36; VAS = Visual Analog Scale.

Among the included studies, five compared DMT with control conditions involving no intervention or waitlist. Seven studies evaluated DPA compared with no‐intervention, waitlist, or educational control groups. DPA interventions included four belly dance studies, one square dance study, one Greek traditional dance study, and one ballroom dance study. It is worth noting that most DPA and DMT interventions were conducted in controlled rehabilitation settings, with movements carefully designed to target specific functional domains and coordinated with breathing, stretching, or rhythm control to minimize stress on surgical sites. Although many studies did not report precise exercise intensity, programs were generally progressive, delivered in group or supervised formats, and participants were allowed to modify movements according to their individual physical capacities. On‐site dance therapists and research personnel monitored sessions in real time to ensure safety and prevent injury.

Across studies, 686 women with breast cancer participated (320 intervention; 366 control), aged 35–80 years. Although no staging restriction was applied during selection, all included samples consisted of non‐metastatic patients (stage 0–III), and most participants were either receiving adjuvant therapy or were in the post‐treatment survivorship phase. Reported intervention protocols ranged from 30 to 180 min per session, 1–10 sessions per week, over durations of 3–24 weeks.

### 3.2 Quality Assessments Results

The methodological quality of the included studies is summarized in Table 2. All studies clearly reported eligibility criteria, conducted between‐group statistical comparisons, and employed intention‐to‐treat analysis, achieving overall scores indicative of good quality (5–8 points). Baseline comparability was adequately addressed in 11 studies, and eight studies provided sufficient details on the generation of randomized sequences. However, only two studies implemented participant blinding, and only one study reported assessor blinding. Furthermore, three studies were noted to have inadequate follow‐up rates.

**TABLE 2:** Methodological quality of 12 RCTs (PEDro analysis).

Study	Eligibility criteria and source	Random allocation	Concealed allocation	Baseline comparability	Blinding of participants	Blinding of therapists	Blinding of assessors	Adequate follow‐up (85%)	Intention‐to‐treat analysis	Between‐group statistical comparisons	Reporting of point measures and measures of variability	Items 2 to 11 scores
Boing et al. (2018)	⊕			⊕				⊕	⊕	⊕	⊕	5
Boing et al. (2023)	⊕	⊕		⊕					⊕	⊕	⊕	5
Carminatti et al. (2019)	⊕			⊕				⊕	⊕	⊕	⊕	5
Dibbell‐Hope (2000)	⊕		⊕		⊕			⊕	⊕	⊕	⊕	6
He et al. (2022)	⊕	⊕		⊕			⊕	⊕	⊕	⊕	⊕	7
Hiansdt et al. (2021)	⊕			⊕				⊕	⊕	⊕	⊕	5
Ho et al. (2016)	⊕	⊕	⊕	⊕	⊕			⊕	⊕	⊕	⊕	8
Kaltsatou et al. (2011)	⊕	⊕		⊕				⊕	⊕	⊕		5
Leite et al. (2021)	⊕	⊕		⊕			⊕		⊕	⊕	⊕	6
Pisu et al. (2017)	⊕	⊕	⊕	⊕				⊕	⊕	⊕	⊕	7
Rubio et al. (2022)	⊕	⊕	⊕	⊕					⊕	⊕	⊕	6
Sandel et al. (2005)	⊕	⊕	⊕	⊕				⊕	⊕	⊕	⊕	7

### 3.3 Meta‐Analysis Results

#### 3.3.1 QoL

**FIGURE 2:**
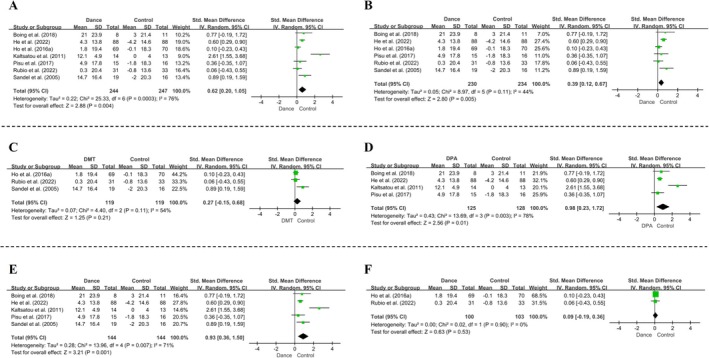
Effects of dance interventions on quality of life in women with breast cancer: Overall and subgroup analyses. (A) Overall forest plot of seven RCTs (*n* = 491) showing standardized mean differences (SMD) and 95% confidence intervals (CI) for QoL outcomes. (B) Sensitivity analyses excluding the Kaltsatou et al. study. Subgroup analyses by intervention type: (C) DMT and (D) DPA; and by intervention duration: (E) ≥ 12 weeks and (F) < 12 weeks.

Seven RCTs (*n* = 491) evaluated the impact of dance interventions on QoL, utilizing the FACT‐B, SF‐36, LSI or EORTC QLQ‐C30 scales. These instruments share comparable scoring systems and domains, with higher scores on functional scales indicating better QoL; positive values for pre‐post changes reflect improvements. The meta‐analysis revealed a significant overall benefit of dance interventions on QoL in women with breast cancer (*SMD* [95% CI] = 0.62 [0.20, 1.05], *p* = 0.004), although substantial heterogeneity was present (*I* ^2^ = 76%, *p* = 0.0003) (Figure 2A). Visual inspection of the forest plot identified Kaltsatou et al. as an outlier, likely due to its unusually long intervention duration (24 weeks) and its effect size differing from the pooled trend. This study contributed only 9% weight to the analysis, suggesting its exclusion would not materially alter results. Sensitivity analysis removing Kaltsatou et al. reduced heterogeneity to 44% (*p* = 0.11) while maintaining statistical significance (*SMD* [95% CI] = 0.39 [0.12, 0.67], *p* = 0.005), supporting the robustness of the findings (Figure 2B).

Subgroup analysis by dance intervention type indicated that DPA significantly improved QoL (SMD [95% CI] = 0.98 [0.23, 1.72], *p* = 0.01) (Figure 2D), whereas DMT did not show a significant effect (SMD [95% CI] = 0.27 [−0.15, 0.68], *p* = 0.21) (Figure 2C). For DPA, removing the Kaltsatou et al. study in a sensitivity analysis reduced heterogeneity from 78% to 0% (*p* = 0.77) and maintained statistical significance (*SMD* [95% CI] = 0.58 [0.31, 0.84], *p* < 0.0001).

We also conducted subgroup analysis based on intervention duration, dividing studies into < 12 weeks and ≥ 12 weeks, as the average intervention duration across studies was 12.4 weeks. Interventions lasting ≥ 12 weeks significantly improved QoL (*SMD* [95% CI] = 0.93 [0.36, 1.50], *p* = 0.001) (Figure 2E), whereas those < 12 weeks did not (*SMD* [95% CI] = 0.09 [−0.19, 0.36], *p* = 0.53) (Figure 2F). For interventions ≥ 12 weeks, sensitivity analysis excluding the Kaltsatou et al. study reduced heterogeneity from 71% to 0% (*p* = 0.75) and preserved statistical significance (*SMD* [95% CI] = 0.62 [0.37, 0.87], *p* < 0.00001).

#### 3.3.2 Depression

**FIGURE 3:**
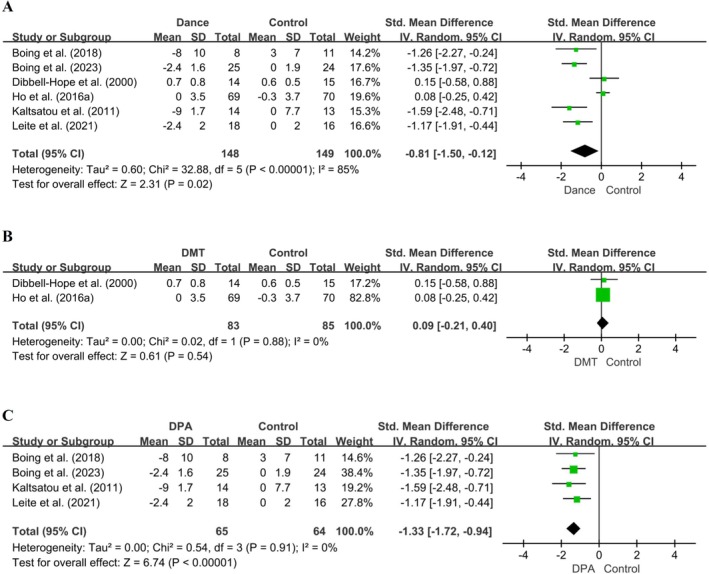
Effects of dance interventions on depression in women with breast cancer: Overall and subgroup analyses. (A) Overall forest plot of six RCTs presenting standardized mean differences (SMD) and 95% confidence intervals (CI) for depression outcomes. Subgroup analyses by intervention type and duration: (B) DMT (< 12 weeks) and (C) DPA (≥ 12 weeks).

Six studies evaluated the impact of dance interventions on depression in women with breast cancer using the BDI, the SCL‐90 depression subscale, and the HADS. For all instruments, higher scores indicate more severe depressive symptoms; therefore, negative pre–post change values reflect symptom improvement. The meta‐analysis demonstrated a significant reduction in depression following dance interventions (*SMD* [95% CI] = −0.81 [−1.50, −0.12], *p* = 0.02), although heterogeneity was substantial (*I* ^2^ = 85%, *p* < 0.0001) (Figure 3A).

Sensitivity analyses identified (Dibbell‐Hope 2000) and Ho et al. as influential outliers. Both studies employed short‐duration DMT interventions (< 12 weeks) and included predominantly early‐stage (0–I) breast cancer patients. Excluding these studies reduced heterogeneity to 0% while preserving a significant pooled effect (*SMD* [95% CI] = −1.33 [−1.72, −0.94], *p* < 0.0001), supporting the robustness and reliability of the overall findings (Figure 3C).

Subgroup analyses further corroborate this rationale: DMT interventions < 12 weeks did not significantly affect depressive symptoms (*SMD* [95% CI] = 0.09 [−0.21, 0.40], *p* = 0.54) (Figure 3B), whereas DPA interventions ≥ 12 weeks produced substantial reductions (*SMD* [95% CI] = −1.33 [−1.72, −0.94], *p* < 0.0001) (Figure 3C). Thus, the exclusion of these two studies for sensitivity analysis is justified not by statistical convenience but by their divergence in therapeutic modality and insufficient duration to elicit measurable effects, ensuring that the pooled estimate accurately reflects the influence of sustained dance‐based interventions on depressive symptoms.

#### 3.3.3 Fatigue, Pain and Body Image

**FIGURE 4:**
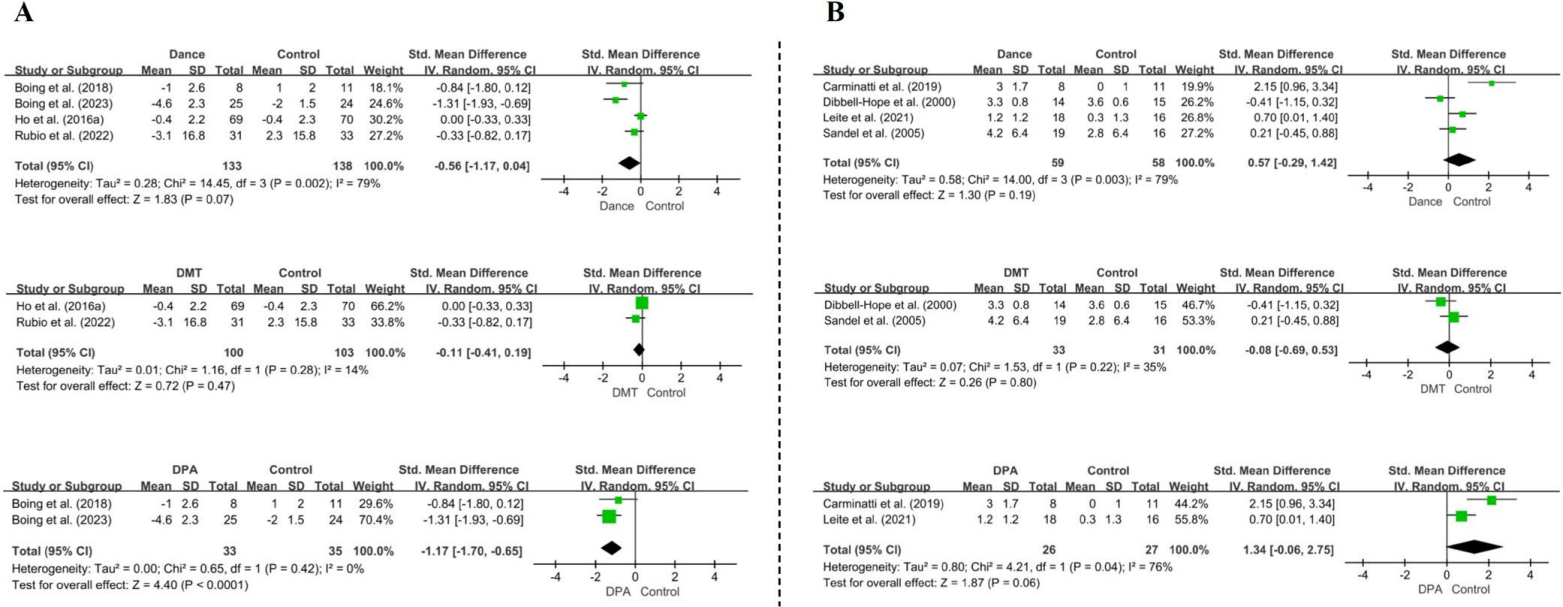
Effects of dance interventions on fatigue (A) and body image (B) in women with breast cancer: Overall and subgroup analyses. In each panel, the overall forest plot is shown at the top, followed by subgroup analyses according to intervention type (DMT vs. DPA).

Four RCTs (*n* = 271) assessed fatigue using PFS, BFI, and FACT‐Fatigue scales, with higher scores indicating more severe fatigue. No significant difference was found between the dance and control groups (*SMD* [95% CI] = −0.56 [−1.17, 0.04], *p* = 0.07), with substantial heterogeneity (*I* ^*2*^ = 79%, *p* = 0.002). However, subgroup analysis confirmed that DMT interventions lasting < 9 weeks did not significantly affect fatigue (*SMD* [95% CI] = −0.11 [−0.41, 0.19], *p* = 0.47). In contrast, the remaining two studies using dance‐based physical activity (DPA) with durations ≥ 9 weeks demonstrated a significant reduction in fatigue (*SMD* [95% CI] = −1.17 [−1.70, −0.65], *p* < 0.0001) (Figure 4A).

Four RCTs assessed body image using the BIABCQ, BWB, EAR, and SF‐36 scales. The pooled analysis showed no significant effect of dance interventions on body image (*SMD* [95% CI] = 0.57 [−0.29, 1.42], *p* = 0.19), with substantial heterogeneity (*I* ^2^ = 79%, *p* = 0.003). A random‐effects model was therefore applied. Subgroup analysis based on intervention duration was not feasible because only one study lasted 6 weeks, whereas the remaining studies exceeded 12 weeks. Consequently, studies were stratified by intervention type. No significant effects were observed for either DMT (*SMD* [95% CI] = −0.08 [−0.69, 0.53], *p* = 0.80) or DPA (*SMD* [95% CI] = 1.34 [−0.06, 2.75], *p* = 0.06) (Figure 4B).

**FIGURE 5:**
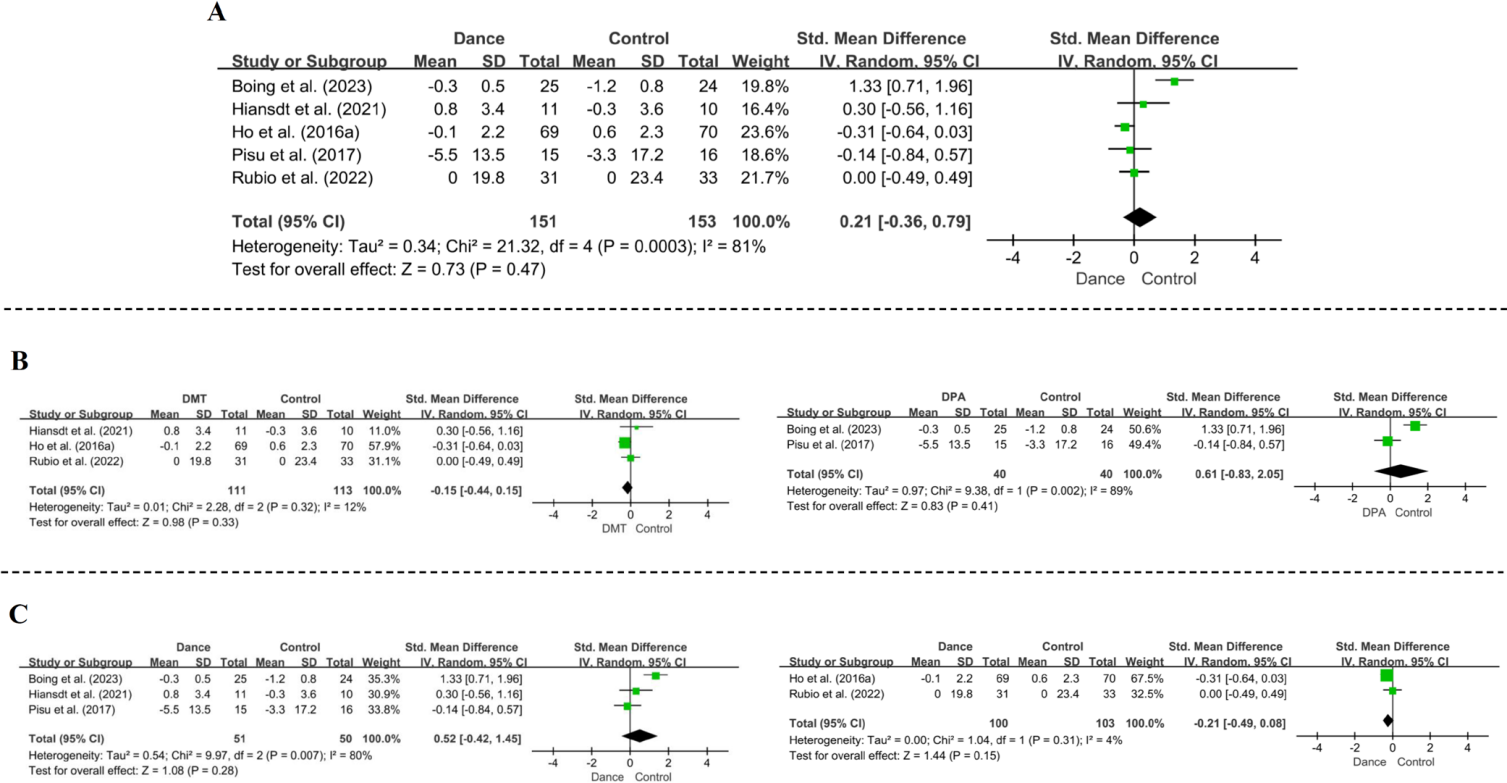
Effects of dance interventions on pain in women with breast cancer. (A) Overall forest plot. (B) Subgroup analysis by intervention type (DMT vs. DPA). (C) Subgroup analysis by intervention duration.

Five RCTs evaluated pain outcomes. The pooled analysis showed no significant effect of dance interventions on pain (*SMD* [95% CI] = 0.21[−0.36, 0.79], *p* = 0.47), with considerable heterogeneity (*I* ^2^ = 81%, *p* = 0.0003) (Figure 5A). To explore potential sources of heterogeneity, studies were stratified by intervention duration using 10 weeks as the cutoff. Subgroup analysis showed no significant effects for either < 10 weeks (*SMD* [95% CI] = −0.21[−0.49, 0.08], *p* = 0.15) or ≥ 10 weeks (*SMD* [95% CI] = 0.52 [−0.42, 1.45], *p* = 0.28) (Figure 5C). Similarly, stratification by intervention type revealed no significant effects for DMT (*SMD* [95% CI] = −0.15 [−0.44, 0.15], *p* = 0.33) or DPA (*SMD* [95% CI] = 0.61 [−0.83, 2.05], *p* = 0.41) (Figure 5B).

## 4 Discussion

The primary aim of this meta‐analysis was to determine whether dance‐based interventions improve psychosocial outcomes in breast cancer survivors and whether effects differ according to therapeutic orientation and intervention duration. Across pooled analyses, dance interventions were associated with significant improvements in quality of life and reductions in depressive symptoms. However, these benefits were not uniformly distributed across modalities. Subgroup analyses indicated that effects were predominantly driven by DPA rather than DMT, particularly when intervention duration exceeded 12 weeks. For fatigue, no overall between‐group difference was detected, yet DPA interventions lasting at least 9 weeks produced significant reductions, whereas DMT showed no comparable effect. In contrast, neither pooled nor subgroup analyses demonstrated meaningful improvements in pain or body image. Collectively, these findings suggest that treatment response is moderated by both therapeutic orientation and intervention dose rather than by the general category of dance intervention alone.

### 4.1 Effects of Dance Interventions on QoL and Psychological‐Related Factors

Dance interventions significantly enhance QoL and alleviate depression symptoms in women with breast cancer. QoL is a multidimensional construct often negatively affected by depression, which persists in up to 25% of breast cancer survivors even 5 years post‐diagnosis (Bedillion et al. 2019). Our meta‐analysis demonstrated robust improvements in QoL and reductions in depression following dance interventions, with heterogeneity becoming nonsignificant after removing outlier studies. Subgroup analyses revealed that these benefits were primarily driven by DPA rather than DMT, particularly when intervention duration exceeded 12 weeks.

These results align with prior systematic reviews. For example, Bradt et al. (2015) reported limited evidence for the effectiveness of DMT in reducing depressive symptoms, based on a small number of trials. In contrast, belly dance—deeply rooted in traditional Arabic music—has been shown to affirm feminine identity and enhance mental resilience among women coping with a breast cancer diagnosis (Boing et al. 2023). Our meta‐analysis further indicates that DPA interventions—including belly dance, square dance, and traditional Greek dance—emphasize structured movement repetition and motor engagement within group contexts, rather than therapist‐guided emotional processing or creative improvisation. Interventions lasting ≥ 12 weeks appear to confer multiple interrelated benefits.

First, repeated movement sequences in partner and group‐based activities facilitate motor imitation and mirroring, enabling nonverbal communication of emotions, promoting social cohesion, and fostering shared experiences in supportive environments (van Baaren et al. 2009; Christensen et al. 2017; Karpati et al. 2015). Second, synchronization‐intensive movement enhances emotional regulation by allowing participants to resonate with the affective states of peers or instructors through automatic mimicry and observation, thereby improving empathy, emotional processing, and interpersonal connection (Koehne et al. 2016; Wu et al. 2023; Foster Vander Elst et al. 2023; Teixeira‐Machado et al. 2019). Third, DPA may support psychological recovery via physiological mechanisms, including neuroendocrine regulation, stress reduction, and stabilization of autonomic functions such as heart rate and respiration (Christensen et al. 2017). Collectively, these psychosocial and physiological processes highlight the multidimensional therapeutic value of DPA for breast cancer survivors.

By contrast, DMT targets emotional expression and psychological processing through symbolic and relational movement, emphasizing bodily awareness and improvisation to help participants reconnect with their bodies and gain insight into internal emotional states (Bradt et al. 2015; Ho et al. 2020). DMT relies heavily on the therapeutic relationship and may require longer intervention durations to achieve optimal effects. In the present meta‐analysis, all included DMT interventions lasted less than 10 weeks, which may have constrained efficacy. Accordingly, future randomized controlled trials should directly compare DPA and DMT using matched intervention doses and systematically vary intervention duration to clarify modality‐specific effects and elucidate the biopsychosocial mechanisms underlying dance‐based interventions in oncological care.

### 4.2 Effects of Dance Interventions on QoL and Physical‐Related Factors

Breast cancer patients commonly experience physical discomforts such as fatigue, pain, and impaired body image, all of which substantially influence QoL. These symptoms frequently arise as adverse consequences of cancer treatment. Cancer‐related fatigue is particularly prevalent yet poorly understood, whereas chronic pain is often associated with altered pain inhibition and heightened sensitivity (Zhang et al. 2015). Changes in body image further contribute to dissatisfaction with appearance and poorer QoL outcomes (Fiser et al. 2021). Together, these physical sequelae interact with psychological factors and form an integral component of survivorship burden.

Despite the psychological benefits observed following dance interventions, the present meta‐analysis did not demonstrate significant overall effects on pain, fatigue, or body image. Subgroup analyses by intervention type and duration likewise revealed no consistent improvements across physical outcomes, except for a reduction in fatigue within the DPA subgroup, which was based on a limited sample and should therefore be interpreted cautiously.

One likely explanation relates to intervention dose. Physical outcomes such as fatigue and pain are sensitive to the frequency, intensity, and duration of activity, yet these parameters varied markedly across studies. For example, some trials implemented short programs lasting only a few weeks with relatively low weekly exposure (Ho et al. 2016), whereas others applied substantially longer protocols with much higher cumulative training time (Pisu et al. 2017). This variability suggests that, although dance constitutes a form of physical activity, many interventions may not have reached the physiological loading threshold required to modify persistent treatment‐related symptoms. The limited number of comparable studies and the multidimensional differences in intervention design also prevented more detailed stratified analyses of these parameters.

From a mechanistic perspective, dance—whether DMT or DPA—integrates movement with emotional and social engagement and therefore readily influences psychosocial functioning. In contrast, fatigue, chronic pain, and body image disturbance in breast cancer survivors often reflect long‐term physiological alterations induced by surgery, endocrine therapy, or systemic treatment. Such conditions typically require sustained and sufficiently intensive physical stimulation to induce measurable biological adaptation. Accordingly, the absence of consistent physical improvements in the present analysis should not be interpreted as evidence that dance is ineffective for somatic symptoms, but rather that the therapeutic threshold for these outcomes may not have been consistently achieved in the available studies.

Evidence from broader physical activity research further indicates that improvements in physical functioning depend strongly on exercise dosage and baseline health status (Drewnowski and Evans 2001; Penedo and Dahn 2005). Clinical populations frequently require carefully calibrated intensity and duration to obtain physiological benefits. Future trials should therefore standardize and experimentally manipulate intervention dose parameters—frequency, intensity, and duration—and directly compare different dance modalities to determine the level of exposure necessary to meaningfully affect fatigue, pain, and other physical outcomes in breast cancer survivors.

## 5 Limitations

This review provides a comprehensive synthesis of the current evidence regarding the effects of dance interventions on QoL‐related psychological and physical outcomes in breast cancer survivors; however, several limitations should be acknowledged. First, only English‐language randomized controlled trials were included. In addition, many studies involved small samples and limited participant diversity, which may reduce the external validity of the findings. Second, subgroup analyses across specific DPA styles were not feasible because of the small number of comparable trials within each category. Consequently, the present analysis cannot determine whether the observed effects are consistent across different dance forms. Finally, physical outcomes were primarily evaluated using subjective measures; objective indicators—such as energy expenditure (e.g., kcal), body fat percentage, BMI, or body weight—were rarely reported and may provide a more precise assessment of the physiological impact of dance interventions. Future research should therefore recruit larger and more diverse populations and adopt standardized intervention and reporting protocols, as well as incorporate objective physiological measures, to enhance comparability and strengthen the generalizability of conclusions.

## 6 Conclusions

Dance is a multidimensional intervention integrating physical activity, emotional expression, and social engagement, and appears to offer benefits for quality of life and psychological well‐being in women with breast cancer. The present findings suggest improvements in psychological outcomes, whereas evidence for alleviating physical symptoms remains limited and variable across studies.

For clinical implementation, dance programs should be delivered within rehabilitation settings through collaboration between healthcare professionals and certified dance movement therapists or appropriately trained dance instructors. Hospitals and oncology rehabilitation centers may incorporate structured group‐based sessions supervised by qualified personnel to ensure safety, therapeutic consistency, and sustained adherence. Healthcare providers can support participation by screening patient suitability, tailoring modality choice, and integrating programs into survivorship care pathways.

Future studies should establish standardized protocols regarding intervention dose (frequency, intensity, and duration) and evaluate long‐term adherence in real‐world clinical environments. In addition, incorporating objective outcome measures—such as neuroimaging or other physiological biomarkers—may help clarify mechanisms and strengthen the clinical evidence base. Comparative trials against conventional exercise rehabilitation are also required to determine the specific therapeutic value of dance‐based interventions in breast cancer care.

## Funding

This work was supported in part by the Scientific Research Project of Beijing Educational Committee (no. KM202210051001; no. 0624108/035) to Tingting Chen, as well as by the National Natural Science Foundation of China (No. 32371132) to Changhao Jiang.

## Conflicts of Interest

The authors declare no conflicts of interest.

## Data Availability

The data that support the findings of this study are available from the corresponding author upon reasonable request.
